# Treatment of Nœvi Materni

**Published:** 1850-07

**Authors:** 


*Treatment of Nœvi Materni.* By T. B. Curling, Esq.—The treatment applicable to nævus must be adapted to its form, according as the growth is cutaneous or the bright scarlet kind; sub-cutaneous, or a livid, puffy swelling; or mixed, partaking of the characters of the cutaneous and subcutaneous. Although their external appearance differs considerably, the true structure of the several kinds of nævus is essentially the same. In the cutaneous, the progress of the disease is comparatively slow, owing to the resisting texture of the skin; but this part is so much involved that it is seldom possible to obliterate the growth without the destruction of a superficial layer of the cutis and the formation of a glistening scar. In some instances, after inflammation in the part, or more rarely spontaneously, the bright scarlet appearance fades, and the skin gradually assumes its natural hue. But when a cutaneous nævus is steadily increasing, we must not calculate on this favorable result. In these cases, if the growth be small, and not very prominent, the best mode of getting rid of it is by the application of a powerful escharotic, as the strong nitric acid. If the nævus be of some size, and project, and particularly if it be situated in a part where the areolar tissue is abundant and the skin yielding, as the neck or labium, (which latter is not an uncommon place,) the most effectual plan is to strangulate the growth, either singly, or in two, four or many parts, according to the extent and form of the growth. The removal of a nævus in this way causes a small scar, but the mark is much less less evident than would be expected from the size of the tumor, and after the lapse of a few months, at the growing period, is often barely perceptible. In cases in which the nævus is not adapted for tying, owing to its situation on parts which cannot well be destroyed, or where the skin is adherent, the disease may be extirpated by an operation, I believe suggested by Sir B. Brodie, viz: sub-cutaneous cauterization. A very small knife—a small tenotomy one will answer the purpose—is to be introduced through the sound skin at the side of the tumor, and, being passed into its middle, is to be moved about so as to lacerate the morbid tissue in all directions. A fine-pointed probe, coated with the nitrate of silver, is then to be inserted at the small wound, and freely applied to the lacerated part. The application effectually stops the bleeding, which, if not arrested by the pressure of the finger, is generally profuse. It produces inflammation, which leads to obliteration of the nævus. It sometimes causes ulceration, or a small slough of the part, which more certainly secures the removal of the morbid tissue.

The treatment of sub-cutaneous nævus must be conducted somewhat differently from the preeeding: it spreads more rapidly than the cutaneous, and generally requires to be attacked without delay; yet even this form of nævus is sometimes stationary, and, after remaining in an indolent state for some months, may dwindle into a small puffy swelling of no importance. I have occasionally excised from adults a swelling, the progress of which had thus ceased spontaneously. Sub-cutaneous nævi can often be got rid of without destruction of skin, and consequently without any deformity. The principle upon which this is effected is the excitement of inflammation in the recticular tissue, and its consolidation or obliteration by the effusion of lymph. The chief obstacle to success is the indisposition of this tissue to inflammatory action. It may be freely cut up and otherwise actively treated, without more inflammation being excited than is sufficient to repair the mischief and to stop the spreading of the disease for a brief period. Various methods of exciting inflammation are practised: the injection of stimulating fluids, the introduction of setons; the sub-cutaneous application of the nitrate of silver, &c. The plan of passing numerous setons through the tumor has several advantages: it can be used in all cases and in all situations; its effects can be regulated by the period during which the threads are retained, as well as by moistening them with stimulating liquids; it produces no scar, causes but little suffering, and is a pretty effectual remedy. It occasionally fails, and then other means must be resorted to.

The mixed is a common form of nævus, and the most difficult to treat. The sub-cutaneous portion may be obliterated by the passage of setons, after which the cutaneous will be required to be destroyed by escharotics; but as this double process involves a destruction of skin and the formation of a scar, I generally prefer, if the tumor be not of considerable size, having recourse at once to the ligature, as the most certain and effectual treatment. A mixed nævus, situated on the face, may sometimes be advantageously removed by the sub-cutaneous ligature. In this mode a strong ligature is carried around the base of the tumor, immediately beneath the sound skin at its border, and strangulation of the growth is effected without slough or destruction of the integuments. The sub-cutaneous part of the nævus is effectually destroyed by inflammation and suppuration, and the passage of blood into the cutaneous portion being in a great degree intercepted by constriction of the tissue beneath, this fades and disappears, whilst the nourishment which it receives from the circulation in the adjoining skin prevents it from perishing. This mode of applying a ligature is applicable to many cases of simple sub-cutaneous nævus. It is less painful than, and in other respects preferable to, the plan sometimes adopted, of dividing the skin around the growth before strangulating it with a ligature.—*London Med. Gaz.*

